# Molecular Biomarkers of Neuronal Injury in Epilepsy Shared with Neurodegenerative Diseases

**DOI:** 10.1007/s13311-023-01355-7

**Published:** 2023-03-08

**Authors:** Deepika Negi, Simon Granak, Susan Shorter, Valerie B. O’Leary, Ivan Rektor, Saak V. Ovsepian

**Affiliations:** 1 Faculty of Engineering and Science, University of Greenwich London, Chatham Maritime, Kent, ME4 4TB UK; 2 National Institute of Mental Health, Topolova 748, Klecany, 25067 Czech Republic; 3 Department of Medical Genetics, Third Faculty of Medicine, Charles University, Ruská 87, Prague, 10000 Czech Republic; 4 First Department of Neurology, St. Anne’s University Hospital and Faculty of Medicine, Masaryk University, Brno, Czech Republic; 5 Multimodal and Functional Neuroimaging Research Group, CEITEC-Central European Institute of Technology, Masaryk University, Brno, Czech Republic

**Keywords:** Epileptic seizures, Neurodegeneration, Fluid biomarkers, Neurofilament light chain, Tau protein, Axonal degeneration, Neurogranin

## Abstract

In neurodegenerative diseases, changes in neuronal proteins in the cerebrospinal fluid and blood are viewed as potential biomarkers of the primary pathology in the central nervous system (CNS). Recent reports suggest, however, that level of neuronal proteins in fluids also alters in several types of epilepsy in various age groups, including children. With increasing evidence supporting clinical and sub-clinical seizures in Alzheimer’s disease, Lewy body dementia, Parkinson’s disease, and in other less common neurodegenerative conditions, these findings call into question the specificity of neuronal protein response to neurodegenerative process and urge analysis of the effects of concomitant epilepsy and other comorbidities. In this article, we revisit the evidence for alterations in neuronal proteins in the blood and cerebrospinal fluid associated with epilepsy with and without neurodegenerative diseases. We discuss shared and distinctive characteristics of changes in neuronal markers, review their neurobiological mechanisms, and consider the emerging opportunities and challenges for their future research and diagnostic use.

## Introduction

Neurodegenerative diseases are characterized by progressive age-dependent death of neurons in the central nervous system (CNS) with related decline of specific brain functions. Two distinctive features make neurons particularly susceptible to degeneration: (1) incompetence for self-renewal and (2) deterioration in homeostatic mechanisms over lifetime [1, 2]. With aging as the main risk factor, neurodegenerative diseases, which include increasingly common Alzheimer’s disease (AD) and associated dementias, Parkinson’s disease (PD), Lewy body dementia (LBD), and several less known conditions, are on the rise, with no cure currently available [3–5]. The lack of therapies is partly due to the shortage of mechanistic data and effective means for early diagnosis, which, if attained, might facilitate medical interventions before the onset of neuronal damage and irreversible functional loss.

**Fig. 1:**
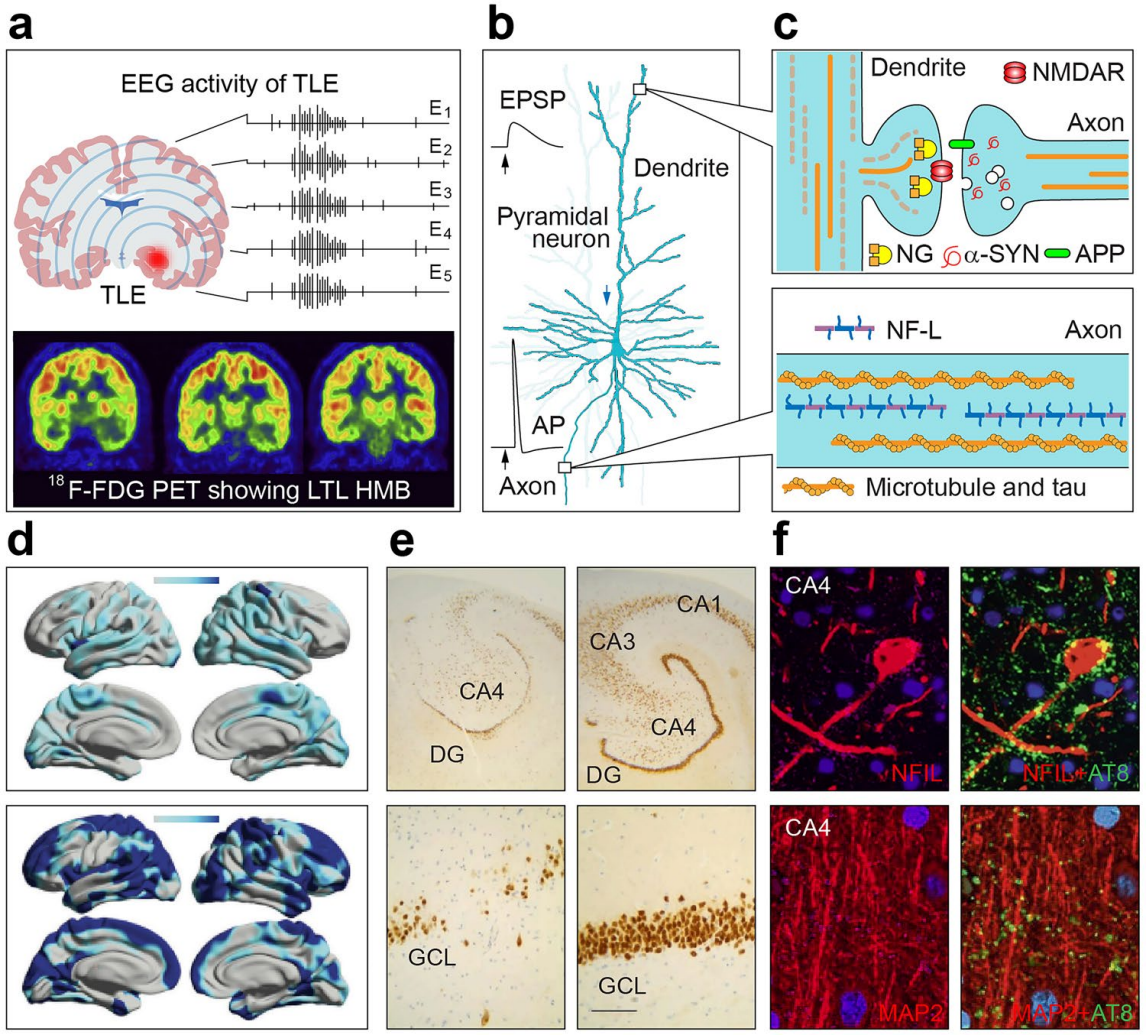
Biomarkers of epilepsy and neuronal proteins. **a**, **d**–**f** Examples of readouts used for diagnosis of epilepsy. **a** EEG and 18F-FDG PET are utilized commonly for detecting super-synchronous electrical activity (seizures) and mapping the location of the epileptic foci based on the level of glucose metabolism, respectively (top and bottom panels). In EEG, epileptic seizures are registered as widespread neuronal synchrony recorded by multiple electrodes (E1-5), while PET imaging shows a reduction in glucose metabolism in the affected brain region, exemplified as left temporal lobe hypometabolism (HMB). TLE—temporal lobe epilepsy. Reproduced with permission from [26]. **d** T1-weighted MRI images comparing cortical thinning in chronic epilepsy: annualized rate in people with epilepsy, with disease duration of more (top) or less (bottom) than 5 years after the onset of the first seizure. Reproduced with permission [27]. **e** NeuN-stained sections of hippocampi from patients with medial temporal lobe epilepsy with and without hippocampal sclerosis (left and right) without and with significant neuronal loss. Scale bars: 1 mm. Reproduced with permission from [28]. **f** Tau accumulation in TLE and hippocampal sclerosis: CA4 neurons labeled with neurofilament light-chain (NF-L) surrounded by pTau/AT8-positive synaptic-like processes in the cell body and axons, or dendritic marker MAP2. Reproduced with permission from [29]. **b**, **c** Schematic representation of physiological expression sites of neuronal proteins found in CSF and blood in epilepsy. *NG* neurogranin, *APP* amyloid precursor protein, *NMDAR N*-methyl-d-aspartate receptor, α-*SYN* α-synuclein, *AP* action potential, *EPSP* excitatory postsynaptic potential

Post-mortem histopathological examination remains the gold standard for differential diagnosis of neurodegenerative diseases (Fig. 1). As the method is applied to brain autopsy, it is of no clinical or therapeutic value to patients. The commonly utilized brain imaging and neurophysiological recordings while highly instructive for revealing anatomical and functional changes are of limited sensitivity for detecting subtle alterations during the early stages of pathology [6–10]. Cortical thinning and atrophy of selected brain regions, for instance, which are taken as imaging biomarkers of brain pathology in AD and LBD, appear at more advanced disease stages, indicating a widespread degeneration of neurons [6, 11–15]. Likewise, changes in neuronal activity detected by electroencephalography (EEG) reflect large-scale impairments of brain connectivity and neural synchrony, inferring a breakdown of long-range connections with disruptions in subcortical modulator influence [16–18]. Over recent years, a rapidly expanding portfolio of molecular biomarkers has shown major promise for the detection of early neurodegenerative processes, which include alterations in a variety of neuronal proteins and their autoantibodies in the blood and cerebrospinal fluid (CSF) of patients [19–23]. These advances have transformed the basic tenets of diagnostics and clinical studies [20, 22, 24, 25], paving a way for new prognostic and treatment opportunities.

Notwithstanding major advantages, molecular biomarkers of neurodegenerative diseases have considerable limitations, which include relative specificity and significant variability of the readouts, partly because of the overt and latent comorbidities [21, 23, 30]. Among the latter, epilepsy is of key interest, partly due to the growing recognition of the effects of seizures on profiles of neuronal proteins in blood and CSF. Given the increasing evidence for alterations in neuronal proteins in biological fluids associated with multiple types of seizures, there is a pressing need in elucidating their origin and interactions with established molecular biomarkers of epilepsy [31–33], as well as characterising their response to antiseizure medications (ASM) [34, 35]. In this study, we review changes in neuronal markers in CSF and blood in epilepsy with and without neurodegenerative diseases. We explore neurobiological mechanisms underlying alterations in molecular biomarkers and consider the emerging opportunities and challenges for their diagnostic and clinical use.

## Molecular Biomarkers of Neurodegenerative Diseases: An Overview

In neurodegenerative diseases, an increase in the level of neuronal proteins and their fragments in CSF and blood are viewed as markers of injury and loss of nerve cells. Due to differential distribution in various neuronal compartments and the specific role played in pathobiology, some of the changes in neuronal proteins can provide additional clues about the type of pathology and the extent of the damage. Alterations in Aβ and tau, for instance, are explored primarily as indicators of amyloid and neurofibrillary tangle pathology and related neuronal loss, while the increase in α-synuclein, neurogranin, synaptic vesicle glycoprotein 2A and synaptosomal-associated protein 25 kD (SNAP-25) are viewed as markers of synaptic damage. The rise in neurofilament light chain (NF-L) level in fluids, on the other hand, is taken predominantly as a marker of axonal injury leading to a collapse of long-range connections [25] (Fig. 1).

In early preclinical and advanced stages of AD, most reports show a notable reduction in the level of Aβ1-42 in CSF and serum, with alternatively cleaved amyloid precursor protein (APP) fragments also reduced compared to age matched controls [36–38]. The level of tau protein increases in the blood and CSF in several neurodegenerative conditions (tauopathies), including AD, frontotemporal dementia (FTD), PD and LBD, amyotrophic lateral sclerosis, and prion disease [30, 39, 40]. In AD and related dementias, total tau and phosphorylated tau (t- and p-tau, respectively) in fluids are significantly higher compared with healthy controls, and correlate with the cognitive decline of patients, with very high levels of t-tau and p-tau in CSF predicting poor clinical outcomes [41–43]. An increase in α-synuclein is regarded mainly with synaptic loss and neuronal degeneration of PD and LBD, with a significant increase of this protein, also detected in biofluids [44, 45]. Like α-synuclein and tau protein, higher levels of NF-L were found in CSF and blood of multiple neurodegenerative diseases, which correlates with axonal breakdown [46–48]. In contrast to α-synuclein and some other proteins enriched in presynaptic terminals (i.e., BACE1 and SNAP-25), neurogranin is a post-synaptic protein of glutamatergic neurons, with its rise in blood and CSF taken as an indicator of damage and loss of dendritic spines [49, 50].

Despite important differences in their molecular pathology and clinical presentation, neurodegenerative diseases share important aspects of their pathobiology, leading to neuronal death. Some neurodegenerative diseases also share comorbidities, which can influence the molecular pathology and profiles of biomarkers. With aging and neurodegenerative disease-related increase in the odds of epilepsy, the impact of seizures on neuronal protein changes has become of major research and clinical interest. In AD, for instance, the risk of seizures can be as high as 35–50% [51–56]. High incidences of seizures were also reported in corticobasal degeneration, LBD, FTD, and PD [52, 54]. As detailed below, several types of epilepsy can alter levels of neuronal proteins in CSF and blood, which warrant comprehensive analysis to decide on their potential effects on profiles of molecular biomarkers of neurodegenerative diseases.

## Aβ and Other APP Fragments in Biofluids of Epilepsy

It has long been known that AD and temporal lobe epilepsy share several histopathological features, including neuronal injury, gliosis, inflammation, and amyloid pathology [57, 58]. Senile plaques have been described in autopsies of patients with epilepsy before the first report of plaques in AD brain samples [59], with higher incidents of age-related amyloid pathology reported in patients with epilepsy than in age-matched controls without epilepsy [60]. Histopathological analysis of amyloid plaques in temporal lobectomy specimens showed both diffuse and neuritic plaques, which were stained with Bodian and Congo red but not with Gallyas methods [60, 61]. These findings implied that despite shared morphology and inclusion of Aβ, senile plaques of epilepsy are devoid of fibrillary tangles.

While the mechanisms for the convergence of histopathological features of AD and epilepsy brain samples remain elusive, growing data imply that neuronal hyperactivity associated with seizures can influence APP processing, as reflected in changes of Aβ isoforms and fragments of soluble APP in CSF [62]. Shahim and co-workers compared levels of Aβx-38, Aβx-40, Aβx-42, Aβ1-42, and sAPP fragments in CSF of patients with single and repetitive generalized tonic–clonic and partial seizures, and non-convulsive status epilepticus (SE) [62] (Table 1). There were no overt differences in Aβ1-42 or sAPP between epileptic patients and controls. However, in patients with repetitive partial seizures, the levels of Aβx-38 and Aβx-40 were higher compared to those with non-convulsive SE, single partial seizures, and controls, while the Aβx-42 was increased in both, patients with single and repetitive partial seizures relative to non-convulsive SE. These findings suggest that the described alterations in APP fragments might result from changes in its processing without neurodegenerative pathology. Interestingly, a comparative analysis of Aβ1-42 and several other neuronal markers in the CSF of AD patients with and without epilepsy showed lower levels of this peptide in AD patients with epilepsy [63]. In the same study, levels of p-tau and t-tau in CSF were enhanced by epileptic activity in AD patients [63]. To investigate if alterations in neuronal proteins of CSF can differentiate prodromal late-onset AD with and without seizures, their variations were quantified and compared [64]. Although the results of this analysis showed no difference, there was a significant correlation of the dynamics of Aβ1-42 and Aβ1-40 in CSF with interictal epileptiform discharges and EEG delta activity. Overall, it emerges that while epileptic seizures *per se* do not alter Aβ1-40 and Aβ1-42 in CSF, in AD patients with epileptic seizures, the level of CSF Aβ1-42 is lower as compared to those without seizures.

**Table 1:** Alterations of Aβ42, Aβx fragments and sAPP in biofluids of epilepsy

Epilepsy type	Age (years)	Gender, N	Fluid	Collection phase	Response to seizures	Ref.
sGTCs, rGTCs, rPS, sPS, nSE	~ 35	F = 12, M = 33	CSF	Post-ictal	Increase (Aβx38,40,42)	[62]
nS, with AD without AD	~ 71, ~ 74	F&M 364	CSF	Inter-ictal	Reduction in Aβ1-42 (in AD with epilepsy)	[63]
fS, rare rGTCS AD or without AD	~ 67, ~ 72	F&M 67	CSF	Post-ictal	Correlation (Aβ40, 42 in AD with epilepsy)	[64]

*sGTCS* single generalized tonic–clonic seizure, *rGTCS* repetitive generalized tonic–clonic seizure, *rPS* repetitive partial seizure, *sPS* single partial seizure, *nSE* non-convulsive status epilepticus, *nS* not specified, *fS* focal seizure

## Tau in Biofluids of Epilepsy

Several reports have shown that the level of t-tau and p-tau is enhanced in the CSF and blood of patients with a single episode of self-limiting [65, 66] or reoccurring seizures [67, 69] (Table 2). Comparison of tau, glial fibrillary acidic protein (GFAP), ubiquitin C-terminal hydrolase-1 and NF-L in blood drawn at baseline, immediately or post 2, 6, and 24 h after a tonic–clonic seizure, demonstrated that all markers were increased postictally and returned to normal levels within hours, with tau changes being most prominent [66]. The authors concluded that both self-limited and reoccurring tonic–clonic seizures can lead to neuronal injury with release of tau protein. Another report addressed the level of tau protein in the blood after acute symptomatic seizures and in poststroke epilepsy (PSE) caused by thrombectomy [70]. No patient included in this study had epilepsy before the intervention, with some of the treated patients developing acute symptomatic seizures while others developed PSE. As the number of patients in this study was low, the results need independent verification in larger cohorts. Tau changes in serum and CSF were analyzed and compared also between patients with autoimmune epilepsy, genetic generalized epilepsy, and psychogenic non-epileptic seizures (PNES) to find if they could assist in differential diagnosis [71]. Neither serum nor CSF tau differed between these groups, implying that tau increments in other studies could involve additional effects associated with seizures.

**Table 2:** Alterations of tau protein in biofluids of patients with epilepsy

Epilepsy type	Age (years)	Gender, *N*	Fluid	Collection phase	Response to seizures	Ref.
TC	~ 32	F = 10, M = 10	Blood	Ictal, Post-ictal, 0 h, 2 h, 6 h, 24 h	Increase, followed by a decrease	[66]
PSE	~ 72	F = 41, M = 49	Blood	Ictal, post-ictal, 0 h, 2 h, 24 h, 48 h, 72 h, 3 months	Increase, followed by a decrease	[70]
nS with AD	~ 63	F&M, 292	CSF	Not specified	Increase (correlated with risk of seizure)	[69]
LOE	~ 70	F = 23, M = 17	CSF	Inter-ictal, post-ictal	Increase	[67]
SE	~ 56	F = 18, M = 10	CSF	Inter-ictal	Increase in refractory SE	[68]
pGE, PE, GCSE, nS	~ 1.40	F = 52, M = 65	CSF	Post-ictal	Increase	[72]

*TC* tonic–clonic, *PSE* post-stroke epilepsy, *nS* not specified seizure, *LOE* late-onset epilepsy of unknown origin, *SE* status epilepticus, *pGE* primary generalized epilepsy, *PE* partial epilepsy, *GCSE* generalized convulsive status epilepticus

Tau profile has been also analyzed in SE using a lumbar puncture, with patients stratified based on their response to ASM, disabilities, and epilepsy outcomes [68]. The levels of t-tau and p-tau were elevated in most of the SE patients, with fluid t-tau values higher in drug-refractory cases compared to those responsive to ASM [68]. In patients with especially high t-tau, the extent of tau increase correlated with the duration of SE. Analysis of the t-tau and p-tau variations and changes in their ratio in CSF collected 48 h after epileptic activity with tonic–clonic or secondary generalized seizures revealed that some patients with acute or remote symptomatic seizures showed altered t-tau levels with p-tau/t-tau ratio differing from controls [65]. As noted earlier, unlike Aβ1-42, the level of t-tau and p-tau in fluids was higher in AD cases with epilepsy [63]. It is interesting to note also that in patients with chronic temporal lobe epilepsy (TLE), the level of t-tau in CSF was lower compared to healthy individuals, while p-tau showed no difference [73]. Measurements of the p-tau/t-tau ratio, on the other hand, yielded higher values in TLE, with changes correlating with the extent of the brain white matter loss. A meta-analysis of children affected by eight different neurological conditions revealed that the concentration of t-tau was significantly increased in epilepsy, similar to that in infectious and inflammatory diseases of the CNS, and showed the highest predictive accuracy for epilepsy and progressive encephalopathy [72]. This report is of special interest as it excludes the effects of aging and related degenerative conditions on dynamics of neuronal and glial proteins in CSF and blood.

## NF-L in Biofluids of Epilepsy

NF-L is another neuronal protein explored as a biomarker of neurodegenerative diseases, with single episodes and reoccurring seizures causing its increase in CSF and blood [74–76] (Table 3). Like studies of tau protein, in blood samples drawn at baseline, immediately after a tonic–clonic seizure and following 2, 6, and 24 h, NF-L was significantly increased after seizures and returned to normal level within several hours [66]. Giovannini and co-workers analyzed changes in NF-L in SE and drug-resistant epilepsy patients and compared them with healthy controls [75]. SE patients showed higher serum NF-L versus patients with refractory epilepsy and controls without epilepsy. Of note, in patients with SE, alterations in serum NF-L showed a stronger correlation with its changes in the CSF, as compared to t-tau protein. The increase in NF-L was more prominent in SE, extending beyond 24 h in refractory/super refractory SE, as well as in patients who died within 30 days, or who presented worsening clinical outcomes. It is worth stressing that NF-L dynamics correlated with ASM response, duration of treatment, and clinical outcomes, offering a potential readout for seizure-related neuronal damage and recovery [75]. An increase in NF-L has been also reported in a study comparing drug-resistant with well-controlled epilepsy and healthy cohort [76]. Analysis of blood samples drawn during the clinical interictal period showed significantly higher levels of NF-L in patients with refractory epilepsy.

**Table 3:** Alterations of NF-L in biofluids of patients with epilepsy

Epilepsy type	Age (years)	Gender, N	Fluid	Collection phase	Response to seizures	Ref.
SS, CE, PSE	~ 53, ~ 51, ~ 72	F = 32, M = 30	Blood	Post-ictal	Increase (higher in PSE & CE vs SS)	[74]
fS, TC	19–59, 19–57	F&M, 89	Blood	Inter-ictal	Increase (higher in DRE vs WCE)	[76]
SE, DRE	~ 45, ~ 39	F = 14, M = 16 F = 13, M = 17	Blood	Post-ictal, 24 h after seizures	Increase (higher in SE vs DRE)	[75]
PSE	~ 72	F = 41, M = 49	Blood	Ictal, post-ictal, 0 h, 2 h, 24 h, 48 h, 72 h, 3 months	Increase	[70]
Focal or diffuse slowing, GS	~ 69	F = 10, M = 14	CFS	Post-ictal	Increase in anti-LGI-1 with epilepsy	[77]
pGE, PE, GCSE, nS	~ 1.40	F = 52, M = 65	CSF	Post-ictal	Increase	[72]
Febrile seizure, nS	~ 2	F = 35, M = 43	Blood	Post-ictal	None response	[78]

*SS* single seizure, *CE* chronic epilepsy, *PSE* post-stroke epilepsy, *fS* focal seizures, *TC* tonic–clonic, *WCE* well-controlled epilepsy, *DRE* drug-resistant epilepsy, *PSE* post-stroke epilepsy, *GS* generalized seizures, *pGE* primary generalized epilepsy, *PE* partial epilepsy, *GCSE* generalized convulsive status epilepticus, *nS* non-specified seizure

A comparative analysis of several neuronal and glial proteins in adults with new-onset self-limiting seizures, chronic epilepsy, and PSE showed significant differences in NF-L between these conditions, with at least 2 years of follow-up demonstrating higher NF-L in PSE vs. self-limiting single seizure cases, and with overall levels of NF-L in patients with chronic epilepsy and PSE exceeding those in patients with single seizure episodes [74]. The same group assessed alterations in NF-L, tau, GFAP, S100, and neuron-specific enolase (NSE) in blood after incidents of symptomatic seizures in patients with a stroke leading to PSE [70]. No patient showed epilepsy before the ischemic stroke. The follow-up time (to death or last medical records review) of 0–4.5 years revealed a 2-year estimated PSE risk of 5.3%. The levels of neuronal and glial proteins in the blood in epileptic patients were above the cohort median. However, the number of PSE cases in this report was small, warranting additional studies. Analysis of neuronal proteins (tau, Aβ42, and NF-L) in CSF were also carried out in patients with anti-leucine-rich glioma-inactivated 1 encephalitis (anti-LGI-1), AD, Creutzfeldt-Jakob’s disease, and primary psychiatric disorders [77]. The concentration of NF-L in AD and anti-LGI-1 encephalitis was comparable and higher than that in psychiatric disorders, but lower than in Creutzfeldt-Jakob’s disease. In anti-LGI-1 encephalitis presenting epilepsy, levels of NF-L were enhanced further compared to cases without epilepsy [77]. Examination of NF-L in CSF of pediatric patients with epilepsy, brain tumor, infectious and inflammatory CNS disorders, static encephalopathy, movement disorders, miscellaneous disorders, and progressive encephalopathy groups with comparison to a control group showed that NF-L was higher in progressive encephalopathy, epilepsy, infectious and inflammatory disorders [72]. Importantly, t-tau, GFAP, and NF-L responded differently to these conditions.

It is interesting to note that in young children (6 months to 5 years), measurements of serum NF-L associated with febrile and epileptic seizures within a few hours after the convulsion revealed no difference as compared to age-matched controls [78]. In multivariable analysis, age was the most important predictor of the rise in serum NF-L, followed by gender and C reactive protein. Studies of the correlation between NF-L changes with the duration of seizures or the time elapsed from seizure onset to blood sampling have revealed no interactions [78]. A similar analysis of the phosphorylated NF-heavy chain (pNF-H) in children showed that prolonged febrile seizures cause a strong increase in serum pNF-H, while brief febrile seizures had no effects [79].

## Other Neuronal Proteins in Biofluids of Epilepsy

Neurogranin and α-synuclein are other two neuronal proteins explored as biomarkers of neurodegenerative diseases. Regulation of calmodulin activity in dendritic spines of glutamatergic synapses makes neurogranin an important molecular switch of synaptic plasticity [80, 81]. Associated with neurodegenerative diseases increase in neurogranin concentration in CSF and blood has therefore been viewed as a specific marker of disruption of synaptic integrity and plasticity. A recent report has shown that neurogranin changes in biofluids can distinguish epileptic seizures from psychogenic non-epileptic seizures (PNES) in adults [82] (Table 4). This carefully conducted study excluded from analysis patients with infectious disease, dementia, stroke, Creutzfeldt-Jacobs’s disease, and brain abscess, as well as patients with a history of traumatic brain injury that could cause neuronal damage or compromise the integrity of the blood–brain barriers. The results of this analysis showed that neurogranin levels in patients with epileptic seizures (confirmed with EEG data) were significantly higher than in PNES and non-epileptic controls [82].

**Table 4:** Alterations of neurogranin and α-synuclein of patients with epilepsy

Epilepsy type	Age (years)	Gender, *N*	Fluid	Collection phase	Response to seizures	Ref.
TC, GS	< 18 years	F = 30, M = 19	Blood	Interictal, post-ictal	NG: increase	[82]
IE, NDE, NIE sGTCS, rPS, sPS	17–68	F&M, 67	CSF, serum	Post-ictal	α-SYN: increase in IE patients	[83]
Afebrile seizure	~ 8.9	F = 47, M = 68	Blood	Post-ictal	α-SYN: increase	[84]

*TC* tonic–clonic, *GS* generalized seizure, *NG* neurogranin, *IE* intractable epilepsy, *NDE* newly diagnosed epilepsy, *NIE* non-intractable epilepsy, *sGTCS* single generalized tonic–clonic seizure, *rPS* repetitive partial seizure, *sPS* single partial seizure

As opposed to neurogranin, α-synuclein is enriched in presynaptic terminals of axons, with evidence suggesting its upregulation in neurons of preclinical models of epilepsy [85]. Clinical data show that α-synuclein level is increased in serum and CSF in children and adults with a history of seizures, which does not respond to ASM [84, 83]. The results of comparative analysis of α-synuclein in serum and CSF of various subgroups of patients indicate that it is increased in cohorts with intractable epilepsy, whereas there was no difference in groups of patients with newly diagnosed or non-intractable epilepsy. These observations led the authors to conclude that the rise of α-synuclein in the serum and CSF may assist in the differential diagnosis of intractable epilepsy [83]. Choi and co-workers compared profiles of α-synuclein and cytokines in serum and exosomes in children with epilepsy and acquired demyelinating disorders of CNS, with readouts assessed against healthy age-matched children. It was found that like in adults, the concentration of α-synuclein and IL-1β were increased in serum and exosomes collected within 48 h after seizures or after relapse of neurological symptoms of autoimmune demyelinating disorders as compared to age-matched controls, and correlated with the severity of epilepsy [84]. Of note, the level of α-synuclein in exosomes is closely related to its level in serum, implying that α-synuclein in both serum and exosomes could help in predicting the severity of children’s epilepsy.

## Release of Neuronal Proteins and Exchange Between CSF and Blood

Although the increase of neuronal proteins in biological fluids of patients with neurodegenerative diseases is viewed primarily in association with the breakdown of neurons and synaptic connections [25, 86], important questions remain over mechanisms underlying their changes related to seizures, given the lack of evidence for neurodegeneration in most types of epilepsy [87–92]. It is tempting to speculate that the bulk of neuronal proteins released during epileptic hyperactivity might go through physiological pathways mediating the secretion of neuropeptides, hormones, and trophic factors from nerve cells [86, 93–95]. Ample evidence also supports the potential involvement of intracellular endosomes in breaking out of proteins from neurons, via exosomes of endosomal origin as well as through budding and fission of the plasma membrane [96–98]. Like in neurodegenerative diseases, in chronic epilepsy, protein-containing endosomes can become abundant and associate with enhanced autophagy and protein secretion pathways [98, 99]. Importantly, the production and release of microvesicles can be promoted by a strong and persistent rise in intracellular Ca^2+^ as well as by cell stress response, which are key characteristics of the pathobiology of epilepsy [100–102]. The prolonged rise of intracellular free Ca^2+^ during intense neuronal activity, in fact, is required for induction of the physiological release of peptides, hormones, and trophic factors from neurons and other secretory cells [93–95, 103]. Finally, associated with epilepsy enhancement of protein degradation mechanisms with release of their fragments in the extracellular space [86, 99, 104] could also contribute to the kinetic and the extent of the variations of neuronal markers in CSF and blood.

It should be noted that in addition to the rate of release from hyperactive and damaged neurons, changes in neuronal proteins in CSF and peripheral circulation may be also influenced by the speed of their exchange between the two compartments, as well as by their degradation by extracellular proteases [105–107]. Along with widely accepted efflux routes of neuronal proteins from the CNS across compromised blood–brain barriers, in epilepsy, they may also escape along the walls of brain arteries and arterioles [106], basement membrane of capillaries [108, 109], as well as through glymphatic drainage routes [110, 111], which are subject to future research.

## Summary and Future Directions

**Fig. 2:**
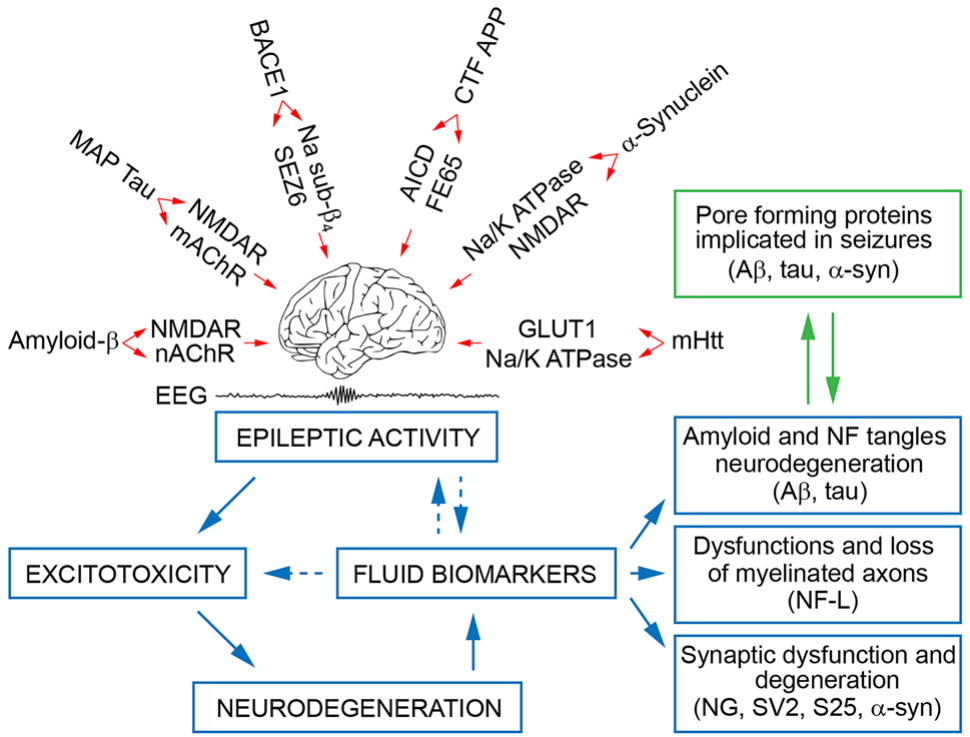
Schematic representation of cause-consequence relation of neuronal injury proteins and epilepsy. In neurodegenerative diseases, several mechanisms contributing to epileptic activity have been described. These include (top, from left to right) activation of NMDAR and nAChR by soluble oligomers of amyloid-β, activation of NMDAR and mAChR by MAP tau protein, cleavage of Na_V_ channel β-subunit by BACE1 protease with dysregulation of Na^+^ channel activity, impairments of Ca^2+^ signalling caused by C-terminal fragment (CTF), and FE65 domain of amyloid precursor protein (APP) released by γ-secretase cleavage, inhibition of Na/K^+^ ATPase, and activation of NMDAR-mediated drive by α-synuclein, and inhibition of glutamate clearance and Na/K^+^ ATPase dysregulation by huntingtin (mHtt) protein [112, 113]. Contributed to these changes epileptic activity leads to the excessive release of synaptic and ectopic glutamate and excitotoxicity [114, 115], which may contribute to neuronal hyperactivity and injury with the release of intracellular proteins. Neuronal proteins explored as biomarkers of neurodegenerative diseases are generally divided as (1) markers of amyloid pathology with neurodegeneration (top), (2) markers of axonal breakdown (i.e., neurofilament light chain, NLF-L), and (3) synaptic degeneration (NG, SV2, and SNAP-25). Amyloid-β, tau, and α-synuclein under pathological conditions can oligomerize and form non-selective cation pores facilitating Ca^2+^ influx with membrane depolarization, which can cause pathological activity in neurons

Epilepsy is among the most prevalent comorbidities of neurodegenerative diseases. Given the global aging population and neurodegenerative diseases on the rise, their intersection with epilepsy has become of major research and clinical interest. Recent data suggest that the incidents of epilepsy in older age and patients with neurodegenerative diseases are widely underrated, mainly due to poor screening and subclinical forms of epilepsy. The mounting clinical evidence implies a complex and dynamic relationship between these morbidities, with both sub-clinical and clinical seizures accelerating the progression and exacerbating outcomes of neurodegenerative diseases (Fig. 2).

Throughout this study, we discussed reports of changes in neuronal proteins in CSF and blood associated with different types of epilepsy with and without neurodegenerative diseases. Based on the dynamics of neuronal markers in fluids and putative release mechanisms of peptides and proteins from neurons, as well as on the lack of evidence for neuronal degeneration in most types of epilepsy, it is reasonable to assume that the transient changes of neuronal markers in CSF and blood related with self-limiting and tonic-clinic seizures could reflect (1) increase in protein metabolism and release from hyperactive neurons, (2) vesicular (exosomal) discharge from stressed and hyperactive neurons, and (3) seizure-related changes in the rate of neuronal protein exchange between CSF and blood. In contrast, the strong and lasting increase of NF-L and tau in SE is likely indicate the extent of the cellular injury with degeneration and associated breakout of neuronal proteins.

With growing recognition of the prevalence of sub-clinical and clinical seizures in the most common neurodegenerative diseases and increasing use of neuronal protein changes as disease biomarkers, addressing the underlying mechanisms and clinical implications are well warranted. Combined with studies of the effects of ASM on dynamics of neuronal proteins and neurodegenerative process, the elucidation of the key mechanistic and translational aspects of neuronal biomarkers shared by epilepsy and neurodegenerative disease are likely to set us on a course to better diagnosis and therapies of these conditions.

## Data Availability

This is a review article and does not contain original data. All questions related to the content of this study should be addressed to the corresponding author.

## Abbreviations

CNS: Central nervous system
AD: Alzheimer’s disease
PD: Parkinson’s disease
LBD: Lewy body dementia
NF-L: Neurofilament light chain
SV2: Synaptic vesicle glycoprotein 2
SNAP-25: Synaptosomal-associated protein 25 kD
CSF: Cerebrospinal fluid
APP: Amyloid precursor protein
FTD: Front-temporal dementia
t-tau: Total tau
BACE1: β-Secretase amyloid cleaving enzyme
sAPP: Soluble amyloid precursor protein
EEG: Electroencephalography
GFAP: Glial fibrillary acidic protein
PSE: Poststroke epilepsy
AME: Autoimmune-mediated epilepsy
PNES: Psychogenic non-epileptic seizures
ASM: Antiseizure medication
TLE: Temporal lobe epilepsy
NSE: Neuron-specific enolase
NF-H: NF-heavy chain
HS: Hippocampal sclerosis
MAP2: Microtubule associate protein 2
NG: Neurogranin
EPSP: Excitatory postsynaptic potential
AP: Action potential
